# Anti-Proliferative, Cytotoxic and Antioxidant Properties of the Methanolic Extracts of Five Saudi Arabian Flora with Folkloric Medicinal Use: *Aizoon canariense*, *Citrullus colocynthis*, *Maerua crassifolia*, *Rhazya stricta* and *Tribulus macropterus*

**DOI:** 10.3390/plants10102073

**Published:** 2021-09-30

**Authors:** Ahmed R. Yonbawi, Hossam M. Abdallah, Faris A. Alkhilaiwi, Abdulrahman E. Koshak, Charles M. Heard

**Affiliations:** 1 School of Pharmacy & Pharmaceutical Sciences, Cardiff University, Cardiff CF10 3NB, UK; YonbawiA@cardiff.ac.uk; 2 Department of Natural Products and Alternative Medicine, Faculty of Pharmacy, King Abdulaziz University, Jeddah 21589, Saudi Arabia; hmafifi@kau.edu.sa (H.M.A.); faalkhilaiwi@kau.edu.sa (F.A.A.); aekoshak@kau.edu.sa (A.E.K.)

**Keywords:** *Aizoon canariense*, *Citrullus colocynthis*, *Maerua crassifolia*, *Rhazya stricta*, *Tribulus macropterus*, anti-proliferative, cytotoxicity, MTT, HaCaT, FACS, live/dead staining, antioxidant, DPPH, RosGlo

## Abstract

Saudi Arabian flora have a history of use as folklore remedies, although such properties have yet to be explored rigorously, and the safety of such remedies should be assessed. This study determined the anti-proliferative, cytotoxic, and antioxidant properties of extracts of the following five plants indigenous to Saudi Arabia: *Aizoon canariense*, *Citrullus colocynthis*, *Maerua crassifolia*, *Rhazya stricta*, and *Tribulus macropterus*. The aerial parts of the five plants were collected from various locations of the western and northern regions of Saudi Arabia and used to prepare methanolic extracts. Three approaches were used to determine the proliferation and cytotoxicity effects using HaCaT cells: MTT, FACS, and confocal microscopy. Meanwhile, two approaches were used to study the antioxidant potential: DPPH (acellular) and RosGlo (cellular, using HaCaT cells). *C. colocynthis* possessed anti-proliferative activity against HaCaT cells, showing a significant decrease in cell proliferation from 24 h onwards, while *R. stricta* showed significant inhibition of cell growth at 120 and 168 h. The IC_50_ values were determined for both plant extracts for *C. colocynthis*, with 17.32 and 16.91 µg/mL after five and seven days of treatment, respectively, and for *R. stricta*, with 175 and 105.3 µg/mL after five and seven days of treatment. *R. stricta* and *M. crassifolia* exhibited the highest capacities for scavenging the DPPH radical with IC_50_ values of 335 and 448 µg/mL, respectively. The subsequent ROS-Glo H_2_O_2_ assay confirmed these findings. The *R. stricta* and *M. crassifolia* extracts showed potent antioxidant activity in both acellular and cellular models. The *C. colocynthis* extract also demonstrated significant anti-proliferation and cytotoxic activity, as did the *R. stricta* extract. These properties support their usage in folk medicine and also indicate a further potential for development for holistic medicinal use or as sources of new active compounds.

## 1. Introduction

Phytomedicine is still widely practiced across the globe due to its low cost and folkloric value in treating a diversity of medical conditions. The search for new treatments based on holistic plant extracts remains a subject of a flourishing research area, as does the hunt for potent lead hits from such extracts. Saudi Arabia is one of the biggest countries in the Middle East, which is reflected in its botanical diversity, as its natural flora provides an abundant, largely untapped source of plant-based drugs with more than 2250 species across 142 families. Furthermore, most of the medicinal plants have not been fully investigated for their pharmacological activities [1,2]. In this paper, we investigated the cell proliferation, cytotoxicity, and antioxidant properties of five plant species indigenous to Saudi Arabia that have a history of purported folkloric medicinal usage.

*Aizoon canariense* belongs to the family of *Aizoaceae*, known locally as Hadag [3], and has been used traditionally to treat hypertension and flatulence [4]. Phytochemical studies of *A. canariense* have revealed the presence of alkaloids, phenolics, triterpenes, coumarins, saponins, tannins, flavonoids, and steroids [4]. *A. canariense* has been shown to possess cytotoxic activity in a brine shrimp bioassay with an LC_50_ of 90.5 μg/mL [5], whereas another study showed cytotoxic activities of extracts of 21.5, 25.7, and 24.5 μg/mL against several cancer cell lines [6]. In antioxidant and antiradical work, the IC_50_ of *A. canariense* was 66 mg/mL using the DPPH assay and 16.06 mg/mL for the ABTS assay [4].

*Citrullus colocynthis* belongs to the family of *Cucurbitaceae* and is known locally as “handhal.” It has been used traditionally for a wide range of conditions, including eye redness, cough, asthma, analgesic, diabetes, jaundice, urinary diseases, ascites, rheumatism, abdominal enlargement, and wound healing [7,8,9]. Previous phytochemical studies on *C.* *colocynthis* seeds and the aerial parts of the plant have shown the presence of fatty acids, tocopherol, and triterpene compounds, particularly cucurbitacins, which have been successfully isolated and identified by several reports. Other studies have demonstrated the presence of glycosides, flavonoids, carbohydrates, alkaloids, and essential oils [9,10]. Unlike other traditional Saudi traditional medicines, this plant has been the subject of several clinical trials. For example, a double-blind clinical trial involving 50 patients diagnosed with type II diabetes found that the plant led to a significant reduction in fasting blood glucose and glycated haemoglobin. However, no noticeable change was reported in the cholesterol profile [11,12,13]. Another clinical trial showed a reduction in the total cholesterol level and triglyceride [14]. The antimicrobial activity of *C.* *colocynthis* was studied by Ali et al. [15] and Bnyan et al. [8], who showed promising results. The cytotoxic activity of alcoholic *C.* *colocynthis* extracts has shown potency against breast and liver cancer cell line lines [16,17]. The antioxidant activity of methanolic fruit extracts has also been evaluated, showing an 88% free radical scavenging activity in a DPPH assay at a concentration of 2500 μg/mL [18].

*Maeruacrassifolia*, belonging to the family of Capparaceae, is known locally as “sarh” or “merro.” Traditionally, it has been used to treat intestinal diseases, gastric ulcers, toothache, and wound healing. Previous phytochemical studies have evaluated the constituents of *M. crassifolia*, revealing the presence of tannins, flavonoids, steroids, cardiac glycosides, alkaloids, saponins, terpenoids, and phenol [19,20]. The plant has antimalarial, anti-inflammatory, analgesic, and antipyretic activities [20,21]. The antibacterial activity of methanolic *M. crassifolia* extract has also been studied, showing potency against a panel of bacteria [22]. A recent study of the antioxidant activity of *M. crassifolia* aqueous extract using the DPPH assay showed free radical scavenging activity of 122 μg/mL [23].

*Rhazya stricta* belongs to the family of *Apocynaceae* and is known locally as “harmal.” It has traditionally been used to treat microbial infections, sore throat, diabetes mellitus, and certain inflammatory conditions such as rheumatism and allergies [7,24,25]. Previous phytochemical studies on *R. stricta* have shown the presence of alkaloids, flavonoids, glycosides, peptides, and triterpenes [26]. Pharmacological studies on *R. stricta* have investigated its anti-inflammatory activity, revealing that the plant extract can increase the production of pro-inflammatory cytokines in mice, possesses antispasmodic and antipyretic activities, and lowers the blood glucose level in rats [27]. *R. stricta* aqueous extract has also been shown to have antimicrobial potency by several works. In one study, the activity towards a *Neisseria meningitidis* isolate, that can cause meningitis, was investigated with a well diffusion assay, showing an inhibition diameter of 9 mm with a concentration of 12 mg/mL, while a microdilution assay reported an MIC of 12 mg/mL [28]. Meanwhile, another study explored the antifungal activity of *R. stricta* using a well diffusion assay [29].

*Tribulus macropterus* belongs to the family of *Zygophyllaceae* and is known locally as “zahr.” Traditionally, it has been used to treat sexual dysfunction and cardiac diseases [30]. Studies on *T. macropterus* constituents have demonstrated the presence of cardiac glycosides, tannins, alkaloids, flavonoids, and saponins [30]. Antihyperglycemic and antihyperlipidemic activity of the plant have been explored, showing promising outcomes [31]. An investigation of the antioxidant activity using a DPPH assay reported a free radical scavenging activity of 42.7% at a concentration of 1 mg/mL [30]. The cytotoxic activity of *T. macropterus* was previously investigated against the cancer cell line HepG2 with an IC_50_ of 2.9 μg/mL [32].

In vitro cytotoxicity and cell proliferation studies form a crucial initial step in indicating the potential toxicity of a test substance, including plant extracts or biologically active compounds isolated from plants, in humans. Minimal toxicity is important for the successful development of a pharmaceutical product, although cytotoxicity may indicate potential value as an anti-cancer agent. Available methods for studying cell proliferation and cytotoxicity were recently reviewed [33]. Each method has limitations with different end-points and it is therefore not possible to provide a universal measure of cytotoxicity because the phenomenon is so complex [34]. Therefore, performing several techniques can provide a more in-depth understanding of the potential toxicity of the five plants being studied, which, to date, have received limited attention and no direct comparison.

In a similar vein, the ability of plant extracts to scavenge ROS was investigated, employing a variety of antioxidant assays, with one of the commonly used being to measure the ability of test substances to scavenge the 1,1-diphenyl-2-picrylhydrazyl (DPPH) radical. This is purely a chemical reaction that is widely used for its simplicity and sensitivity and the production of reliable results [35,36,37]. However, the antioxidant activity in a biological system, such as a live cell, is not comparable due to the range of biochemical processes in progress. Therefore, we used the ROS-Glo H_2_O_2_ assay with the HaCaT cell line to detect anti-ROS activity in cell culture. It is a rapid, sensitive, luminescent assay that measures hydrogen peroxide (H_2_O_2_) levels directly in cultured cells to better identify substances (plant extracts) with ROS scavenging activities.

The aim of this study was therefore to provide a rigorous assessment of extracts of the five selected medicinal plants indigenous to Saudi Arabia in terms of their cytotoxic and anti-proliferative effects towards the HaCaT keratinocyte cell line, and to determine plant antioxidant activities using both acellular and cellular methods.

## 2. Results

### 2.1. Plant Extraction Yields

The aerial parts of *A. canariense*, *C. colocynthis*, *M*, *crassifolia*, *R. stricta*, and *T. macropterus* were extracted with methanol for 72 h via the cold maceration method. The extraction yield represents the amount (solid residue) of plant constituents that were methanol-soluble during the extraction process compared to the weight of plant material before the extraction and is a common extraction approach [38]. The highest extraction yield was obtained with the *R. stricta* extract (12.18% *w*/*w*) and the lowest yield was with the *M. crassifolia* extract (4.43% *w*/*w*). This variation in the yield across different plant extracts can be attributed to the origin of plants, climate, time of collection, and the duration and temperature of the extraction process [39]. Table 1 summarises the yields of the different plant extracts.

### 2.2. Cell Viability Assay

**Figure 1:**
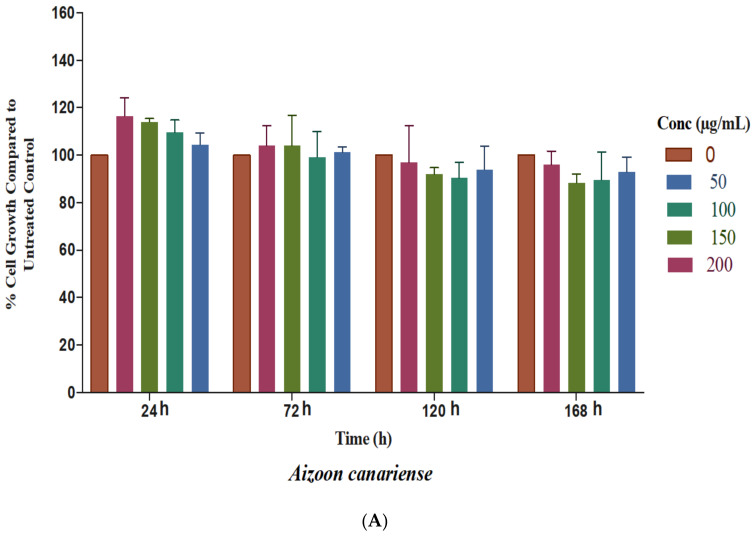

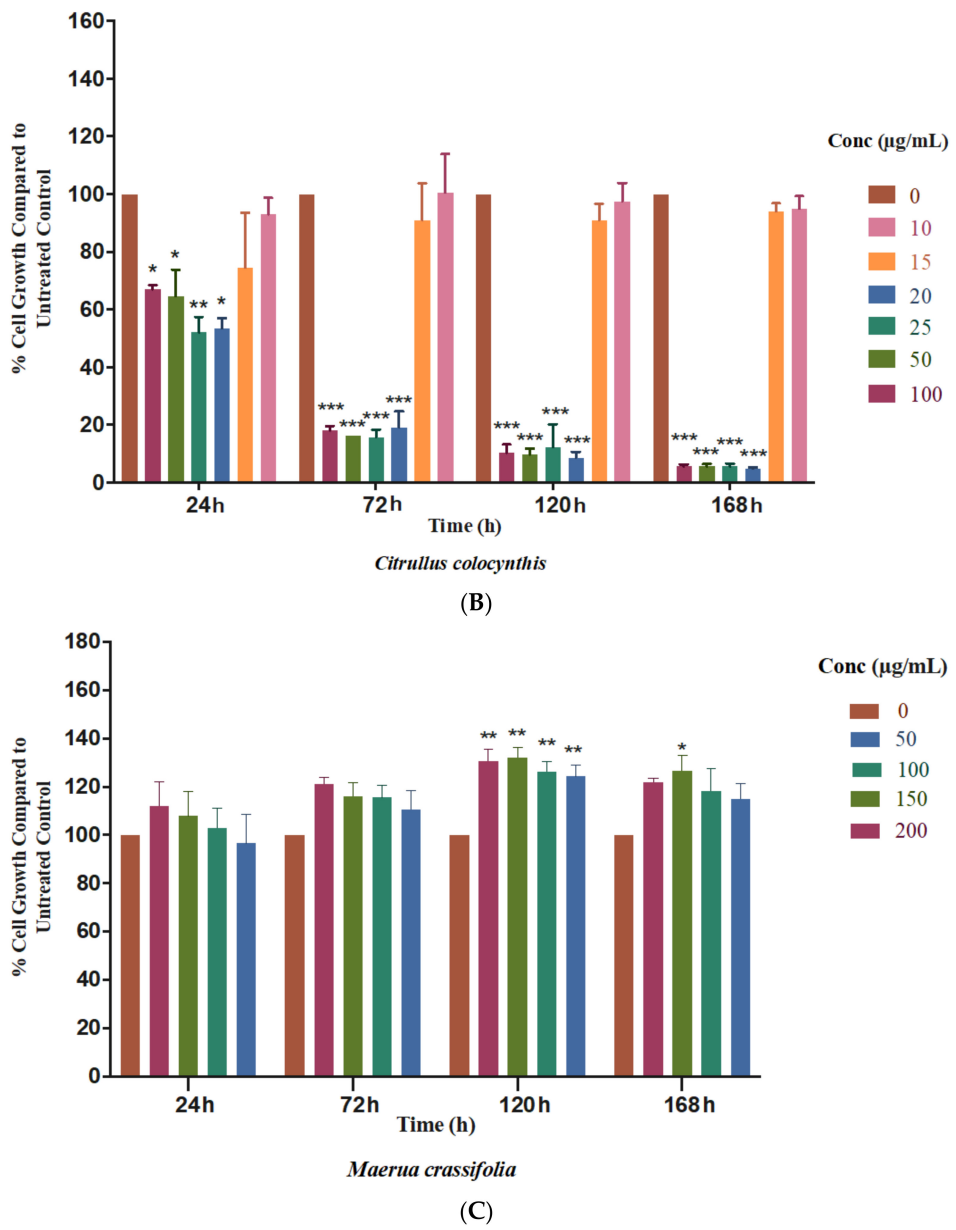

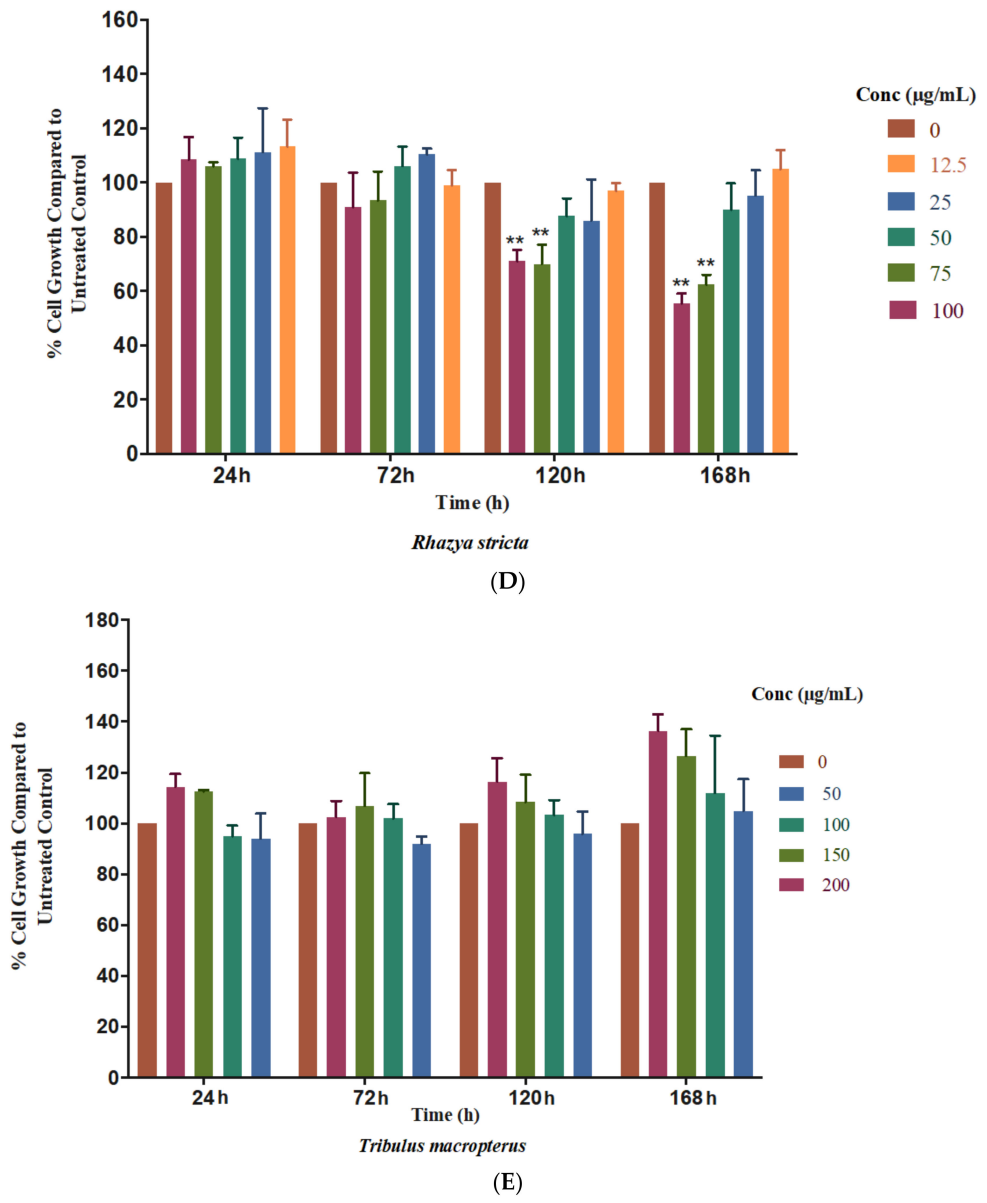
Percentage of HaCaT cell viability and proliferation with (**A**) *Aizoon canariense* extract (50, 100, 150, and 200 µg/mL in the culture medium) (**B**) *Citrullus colocynthis* extract (10, 15, 20, 25, 50, and 100 µg/mL in the culture medium) (**C**) *Maerua crassifolia* extract (50, 100, 150, and 200 µg/mL in the culture medium) (**D**) *Rhazya stricta* extract (12.5, 25, 50, 75, and 100 µg/mL in the culture medium) (**E**) *Tribulus macropterus* extract (50, 100, 150, and 200 µg/mL in the culture medium) at 24, 72, 120, and 168 h compared to the untreated control (mean ± S.D.). The results were obtained through three independent experiments. Statistical analysis of significant changes of the treated cells versus the untreated controls (* *p* < 0.05, ** *p* < 0.01, and *** *p* < 0.001).

The extracts were evaluated at different concentrations: for *A. canariense*, *M. crassifolia*, and *T. macropterus*, the concentrations were 200, 150, 100, and 50 µg/mL; for *C. colocynthis*, the concentrations were 100, 50, 25, 20, 15, and 10 µg/mL; for *R. stricta*, the concentrations were 100, 75, 50, 25, and 12.5 µg/mL. For comparison purposes, untreated controls with vehicle-only (1% methanol, 1% FBS, and DMEM) were included. A three-day MTT assay was carried out and the preliminary results (data not shown) indicated that the 50% inhibition of cell proliferation could not be achieved for several plant extracts, even at a high concentration; thus, the MTT assay was set to be for seven days, and the average absorbance of the extracts at various concentrations was recorded at 24, 72, 120, and 168 h, while the percentage of cell growth from the vehicle-only controls was calculated for each concentration and time point (Figure 1A–E).

Both *A. canariense* and *T. macropterus* showed non-significant effects on HaCaT cell proliferation compared to the control, although the *T. macropterus* extract stimulated the proliferation with concentrations of 200 and 150 µg/mL at 120 and 168 h. Still, this was statistically non-significant (*p* > 0.05). Both plant extracts may be considered to be non-cytotoxic to HaCaT cells.

**Figure 2:**
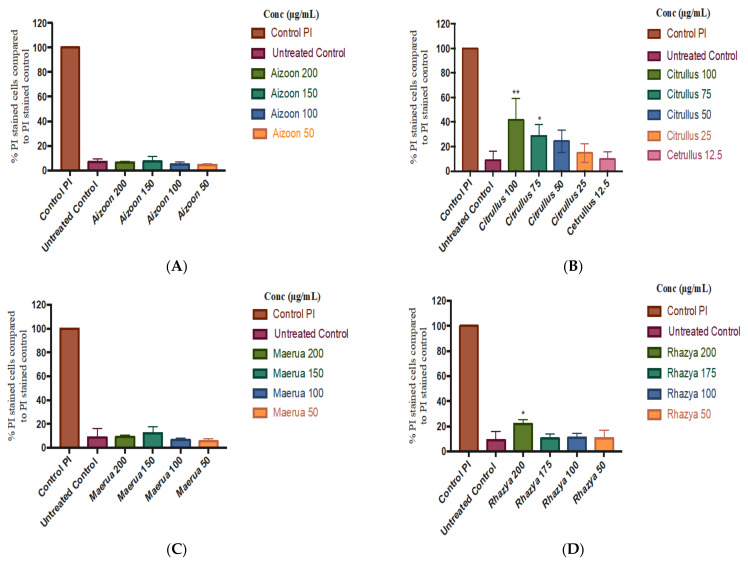

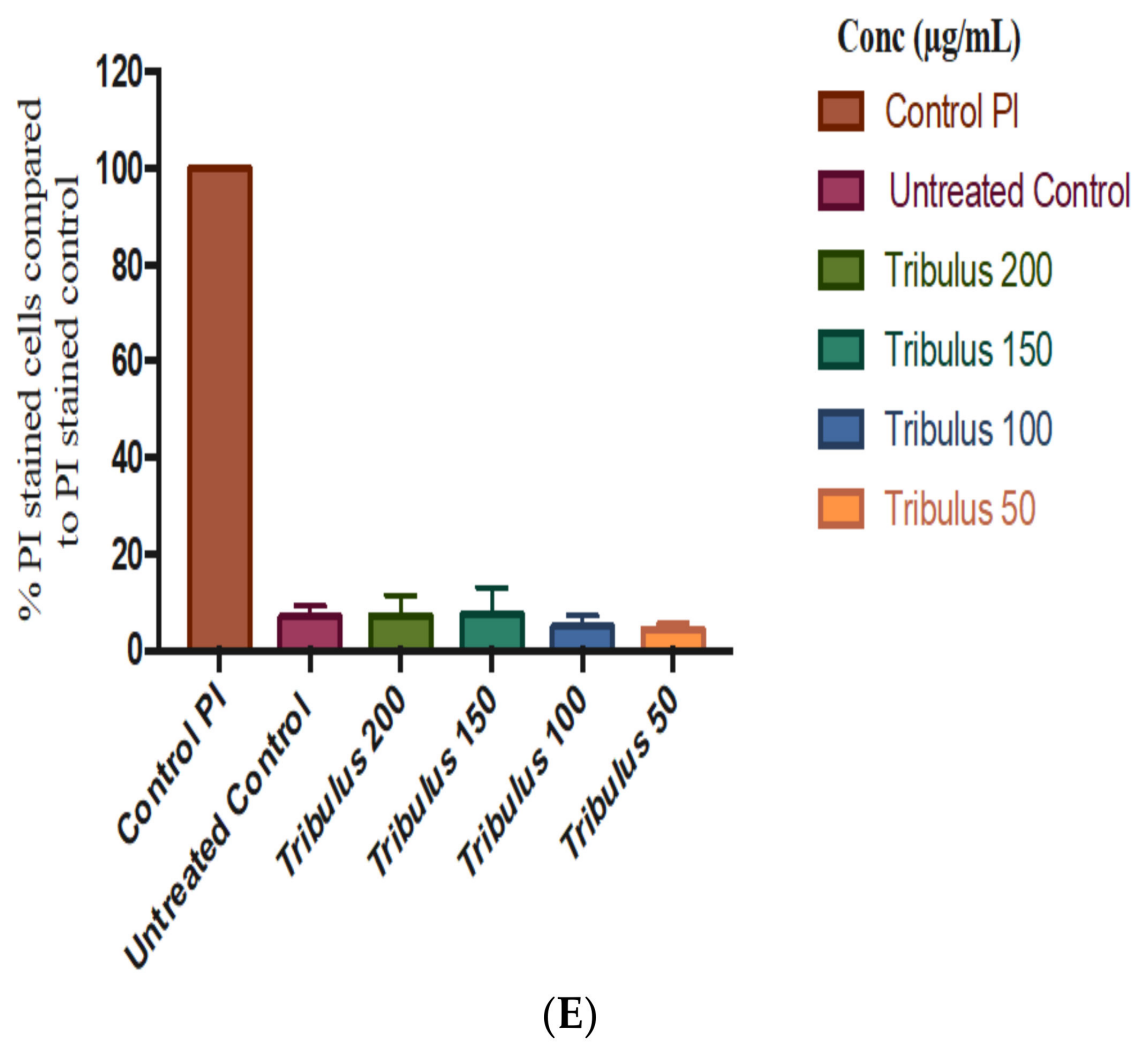
Percentage of HaCaT cells stained with PI for the plant extracts at different concentrations compared to PI control (mean ± S.D.): (**A**) *Aizoon canariense*, (**B**) *Citrullus colocynthis*, (**C**) *Maerua crassifolia*, (**D**) *Rhazya stricta*, and (**E**) *Tribulus macropterus*. The results were obtained through three independent experiments. Statistical analysis of significant changes in the treated cells versus the untreated controls (* *p* < 0.05, ** *p* < 0.01).

Cells treated with *C. colocynthis* at the concentrations of 100, 50, 25, and 20 µg/mL showed a significant decrease in cell proliferation from 24 h onwards, with the most profound inhibition of cell growth at 72 h onwards (*p* < 0.001) (Figure 2B). This indicates that the plant extract may possess cytotoxic activity against HaCaT cells within this range of concentrations (100–20 µg/mL). *R. stricta* showed a delayed but significant inhibition of cell growth at 120 and 168 h at concentrations of 100 and 75 µg/mL (*p* < 0.01) (Figure 1D). The IC_50_ values of *C. colocynthis* and *R. stricta* were calculated from the representation of data on a graph with the best fit line that shows an *R*^2^ > 0.9 (Table 2). *C. colocynthis* exhibited the highest cell proliferation inhibition, with an IC_50_ of 17.32 and 16.91 µg/mL after five and seven days of treatment, respectively. Furthermore, *R. stricta* showed inhibition activity on the last day of the treatment, and the measured IC_50_ values were 175 and 105.3 µg/mL after five and seven days of treatment, respectively.

*M. crassifolia* revealed a significant increase in cell proliferation within the concentration range of 200–50 µg/mL at 120 h (*p* < 0.01), although this stimulation effect continued at 168 h and was deemed to be non-significant (*p* > 0.05), except for cells treated with the concentration of 150 µg/mL (*p* < 0.05) (Figure 1C). This indicates that *M. crassifolia* may possess wound healing potential, which is in alignment with a previous report in its traditional use in wound healing [19].

### 2.3. FACS Analysis

PI/FD double staining FACS analysis was carried out to confirm the cytotoxic properties of the plant extracts found in the MTT assay. The plant extracts were evaluated at the same concentrations as the MTT assay: for *A. canariense*, *M. crassifolia*, *R. stricta*, and *T. macropterus*, the concentrations were 200, 150, 100, and 50 µg/mL; for *C. colocynthis*, the concentrations were 100, 75, 50, 25, and 12.5 µg/mL. The results are presented below (Figure 2A–E).

The cytotoxicity results obtained through the FACS PI/FDA double staining approach were generally comparable to the MTT assay data. *C. colocynthis* at concentrations of 100 and 75 µg/mL showed significant cytotoxic activity towards HaCaT cells after 24 h (*p* < 0.05) (Figure 2B). The data gathered from FACS are considered to be an accurate measurement of cellular damage, and this confirms that the growth inhibition observed in the MTT assay is attributed to the toxicity of *C. colocynthis* towards HaCaT cells rather than cell death. *R. stricta* again demonstrated significant toxicity towards HaCaT cells at the highest concentration of 200 µg/mL after 24 h (*p* < 0.05) (Figure 2D). The other plant extracts showed a similar pattern to MTT results and exhibited no toxicity towards the HaCaT cell line.

### 2.4. Live/Dead Staining by Confocal Laser Scanning Microscopy (CLSM)

**Figure 3:**
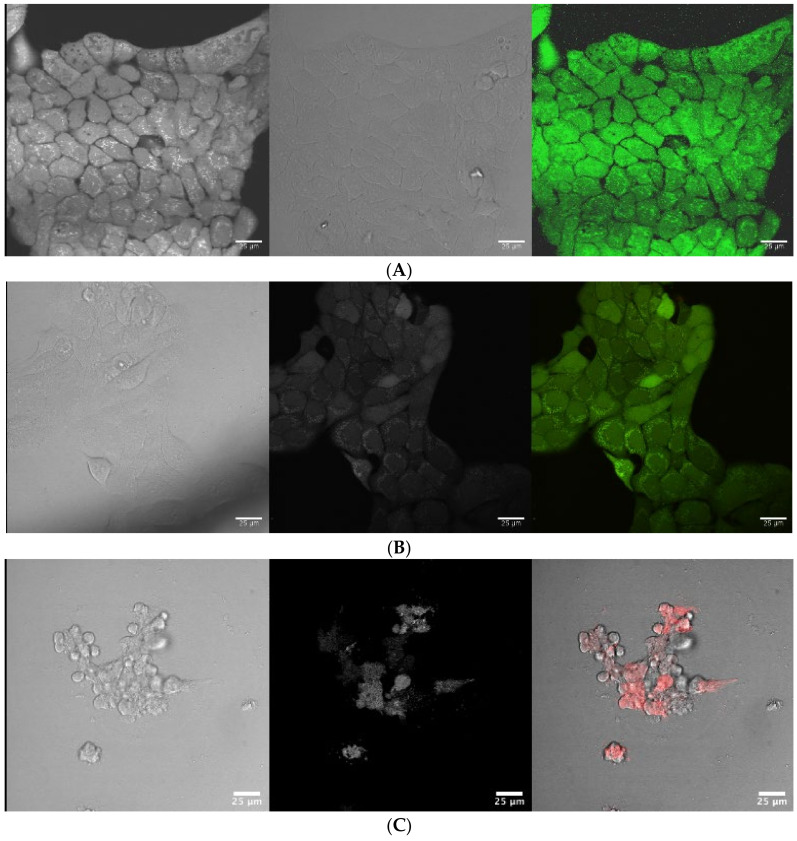

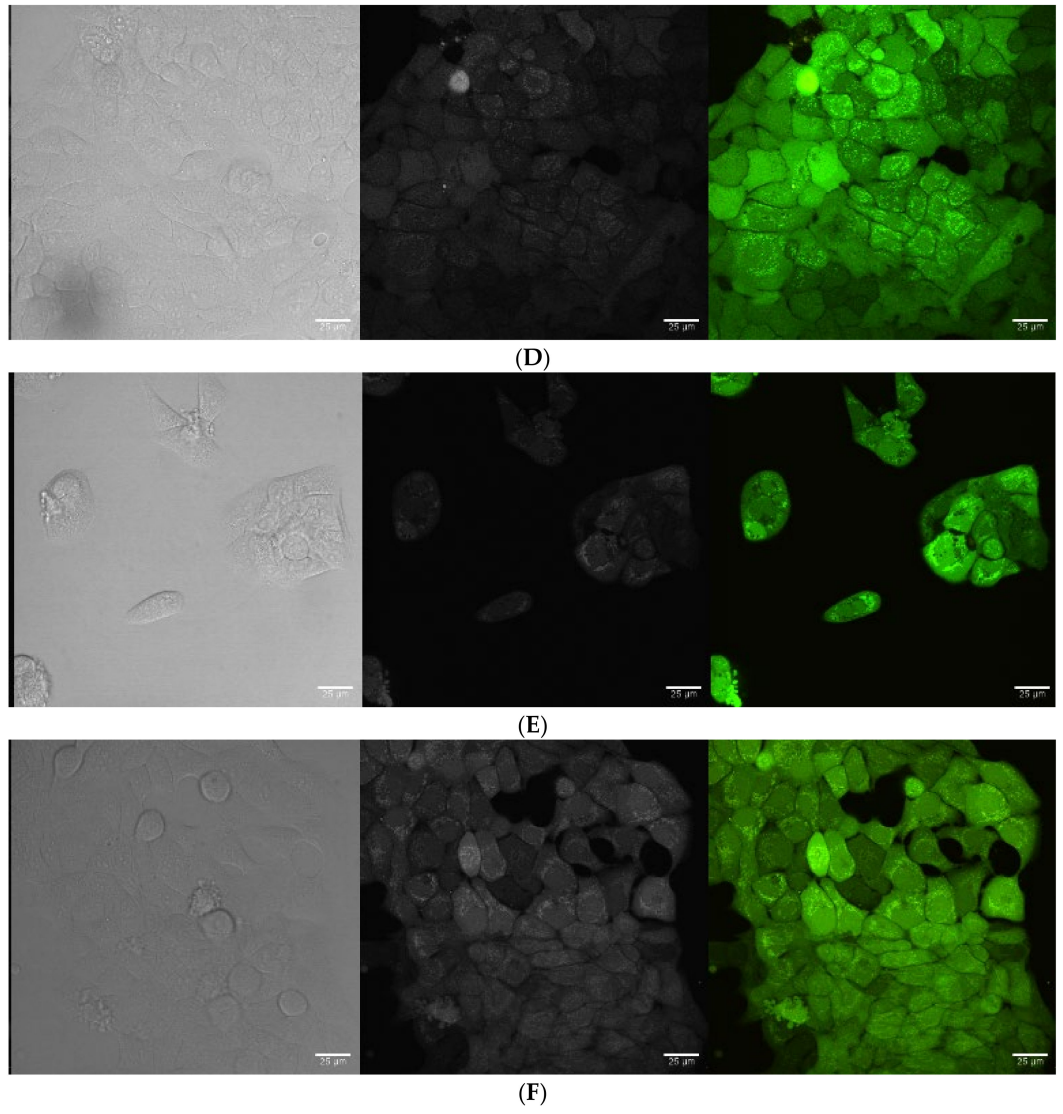
CSLM images of HaCaT cells dual stained with PI and FDA (red and green fluorescent): (**A**) untreated control, where cells appear to be healthy and regularly shaped polygonal cells with high confluency; (**B**,**D**,**F**) cells treated with *Aizoon canariense*, *Maerua crassifolia*, and *Tribulus macropterus*, appearing to be healthy-shaped and homogeneously distributed; (**E**) cells treated with *Rhazya stricta*—although it appears to be healthy cells, confluency was low (around 20%); (**C**) cells treated with *Citrullus colocynthis*, showing extreme morphological changes with very low confluence.

Using confocal microscopy, employing a double staining approach of PI/FD, in FACS analysis, the percentage of PI-stained cells compared to the control plus the percentage of FD-stained cells compared to the control were determined for the plant extracts at the concentrations evaluated above in the MTT assay. Qualitative confirmation of the above viability and cytotoxicity results was obtained by confocal microscopy using the PI/FD double staining method to discriminate between viable and non-viable cells. Plant extracts of *A. canariense*, *M. crassifolia*, *R. stricta*, and *T. macropterus* were examined at the concentration of 200 µg/mL, while *C. colocynthis* was examined at 100 µg/mL (Figure 3A–F).

Generally, the images obtained from CLSM were in line with the viability and cytotoxicity results. The HaCaT cells treated with *A. canariense*, *M. crassifolia*, and *T. macropterus* were stained with FD only, appearing to be healthy and regularly shaped polygonal cells with confluency (up to 90%) and homogeneously distributed without any morphological changes compared to the control. Some of the cells treated with *R. stricta* were stained with FD and appear to be healthy and regularly shaped polygonal cells without morphological changes; however, the confluence was low (around 20%) and some cells were visualised with red PI stain. Cells treated with *C. colocynthis* showed extreme morphological changes with very low confluency, and most cells were stained with PI. *C. colocynthis* demonstrated very high toxicity towards HaCaT cell lines, which complements the other data obtained by MTT and FACS.

### 2.5. Determination of Extracts Total Phenolic Content by Folin Ciocalteu (FC) Assay

A gallic acid calibration curve was utilised to estimate the TFC of five plant extracts presented in Table 3. The highest level of total phenolic content was observed in *Rhazya stricta* which may contribute to the previously stated antioxidant activity, and the lowest level of TFC was observed in *Aizoon canariense.*

### 2.6. DPPH Antioxidant Assay

**Figure 4:**
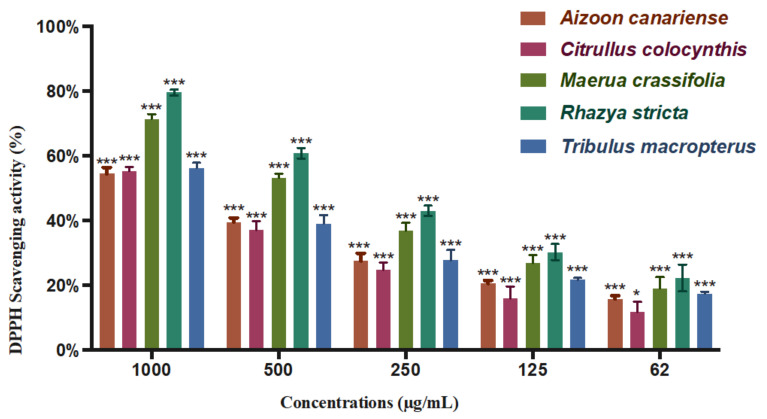
DPPH radical scavenging activity of the plant extracts at different concentrations (µg/mL) (mean ± S.D.). The results were obtained through three independent experiments. Statistical analysis of significant changes compared to the control (* *p* < 0.05, *** *p* < 0.001).

The free radical scavenging activities of the *A. canariense*, *C. colocynthis*, *M. crassifolia*, *R. stricta*, and *T. macropterus* extracts were evaluated in comparison to ascorbic acid through a DPPH assay. DPPH is a stable free radical with a characteristic purple colour. The mechanism of this assay is based on the ability of the potential antioxidant to donate one of its hydrogens to the free radical DPPH, causing a reduction of DPPH to DPPH-H, which will eventually lead to the formation of a yellow colour instead of a purple colour. The DPPH scavenging activities of the different plant extracts are shown in Figure 4.

**Figure 5:**
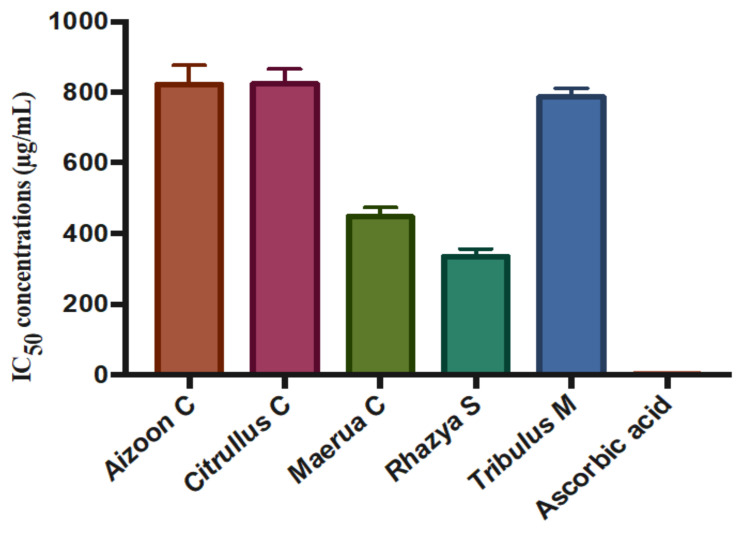
Comparison of the 50% DPPH scavenging activities of the different plant extracts (mean ± S.D.). The results were obtained through three independent experiments.

*R. stricta* and *M. crassifolia* showed the highest capacity for scavenging the DPPH radicals with IC_50_ values of 335 and 448 µg/mL, respectively. These two extracts were capable of eliminating 50% of the harmful radicals at these concentrations. The activity of *R. stricta* can be attributed to the presence of phenolic constituents, such as apigenin, apigenin-8-C-glucoside, quercetin, hesperetin, kaempferol, quercetin-3-rhamnoside, rutin, luteolin, rhazianoside A and B acacetin, and isoquercetin. These compounds have been identified in the plant by several phytochemical studies [26,40]. The antioxidant activity of *M. crassifolia* can be attributed to the presence of phenolic compounds, which have not been identified in phytochemical studies. These results indicate that these two plants present with a high content of phenolic compounds and seem to be a promising source of natural antioxidants that can be utilised to reduce the harmful effects of free radical ROS, which has been linked to many health disorders such as cardiovascular disease, rheumatoid arthritis, several types of cancer, and muscular degeneration. A comparison of the IC_50_ values for the different plant extracts is shown in Figure 5.

### 2.7. ROS-Glo H_2_O_2_ Assay

**Figure 6:**
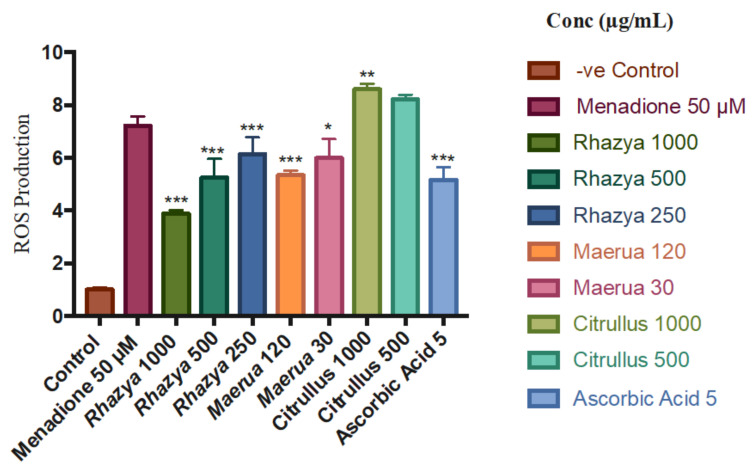
A comparison of the different plant extracts’ ROS production compared to the control and menadione (µg/mL) (mean ± S.D.). The results were obtained through three independent experiments. Statistical analysis of significant changes compared to the control (* *p* < 0.05, ** *p* < 0.01, and *** *p* < 0.001).

**Figure 7:**
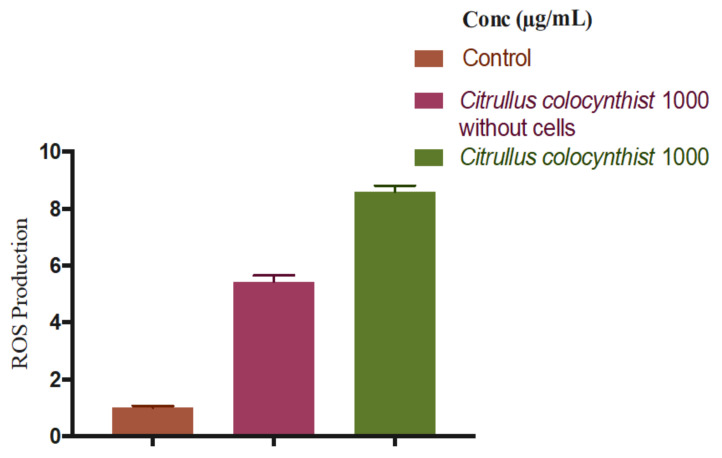
ROS production comparison of the *Citrullus colocynthist* extract control without cells compared to with cells and to the control (mean ± S.D.). The results were obtained through three independent experiments.

Following the DPPH assay, the H_2_O_2_ scavenging activities of *C. colocynthis*, *M. crassifolia*, and *R. stricta* were determined in HaCaT cell culture using a ROS-Glo H_2_O_2_ Assay Kit. In this assay, H_2_O_2_ was detected through the generated luminescence signals and was induced by the addition of menadione; Figure 6 presents a comparison of the different plant extracts’ ROS production inhibition. *R. stricta* showed the highest capacity for ROS radical scavenging with 46, 27, and 14% at the concentrations of 1000, 500, and 250 µg/mL, respectively (*p* < 0.05). Although *M. crassifolia* demonstrated no scavenging activity at the concentrations of 1000, 500, and 250 µg/mL, it displayed some activity at the lower concentrations of 120 and 30 µg/mL with an inhibition percentage of 25% and 16%, respectively (*p* < 0.05). These results are in alignment with DPPH data. On the contrary, *C. colocynthis* at the concentrations of 1000 and 500 µg/mL showed higher ROS production compared to the menadione control (*p* < 0.05). To distinguish between cell-dependent and cell-independent changes in ROS level, the extract control without cells was examined, revealing a higher level of H_2_O_2_. The rationale behind this could be that *C. colocynthis* produces H_2_O_2_ directly or it contains some of the polyphenolic compounds that go through a chemical reaction with constituents of the cell culture media, resulting in the formation of H_2_O_2_. Either way, the cytotoxicity of *C. colocynthis* could be attributed to the excessive production of hydrogen peroxide, which causes cellular damage to HaCaT cells. Figure 7 shows a comparison of the *C. colocynthis* extract control without cells [41].

## 3. Discussion

Folkloric traditional medicines are, by their nature, typically poorly documented, even though they may have a long history of use among the local populace. This work investigated five such plants with medicinal potential in terms of cytotoxic, proliferation, antioxidant activities. Following the collection and extraction process, the HaCaT cell line was utilised to evaluate the anti-proliferative and cytotoxic activity of five plant extracts by the MTT assay followed by FACS analysis with the PI/FD double staining method and qualitative confirmation of viable and non-viable cells with confocal microscopy. Natural products are a source of potential new anti-cancer drugs [42,43,44]. Herein, the results demonstrated that *C. colocynthis* possesses anti-proliferative activity against HaCaT cells, since it showed a significant decrease in cell proliferation from 24 h onwards, while *R. stricta* showed a delayed but significant inhibition of cell growth at 120 and 168 h. The IC_50_ values were determined for both plant extracts for *C. colocynthis*, with 17.32 and 16.91 µg/mL after five and seven days of treatment, respectively, and for *R. stricta*, with 175 and 105.3 µg/mL after five and seven days of treatment.

In the current work, the cytotoxicity results obtained through the FACS PI/FD double staining approach were in alignment with MTT data, *C. colocynthis* showed significant cytotoxic activity towards HaCaT cells after 24 h at the concentrations of 100 and 75 µg/mL. This verifies that the growth inhibition observed in the MTT assay is attributed to the toxicity of *C. colocynthis* towards HaCaT cells rather than cell death. HaCaT cells have been used previously as a model to determine the cytotoxicity of plant extracts [44]. *R. stricta* demonstrated significant toxicity towards HaCaT cells at the highest concentration of 200 µg/mL after 24 h. Images taken by CSLM through the PI/FD double staining procedure served as a quantitative confirmation of the viability and cytotoxicity assay, as it revealed extreme morphological changes of HaCaT cells treated with *C. colocynthis.* These findings indicate that *C. colocynthis* has the potential as a promising source of anti-tumour drug discovery through bioassay-guided fractionation steps. Masuda et al. [45] successfully used flow cytometry to determine the cytotoxic activity in leaf extracts, which led to the isolation of (-)-deoxypodophyllotoxin. In the current paper, the previously unknown cell proliferation activity of the *C. colocynthis* and *R. stricta* extracts may also indicate potential anti-cancer activity.

The free radical scavenging activities of the five plant extracts were evaluated using cellular and acellular models. The damaging effect of reactive oxygen species (ROS) such as hydrogen peroxide, hydroxyl radicals, and superoxide has been linked to many diseases, including cardiovascular disease, cancer, and diabetes. On the contrary, low-level ROS can exhibit a favourable effect in the wound healing process by fighting invading pathogens; however, the excessive production of ROS leads to oxidative damage of the cells and non-healing of wounds [46]. Medicinal plants that possess antioxidant activity can limit the unfavourable effect of ROS through accepting an electron, subsequently forming a more stable product. From the four major plants’ secondary metabolites, alkaloid, flavonoid glycoside, phenolic, terpene, and phenolic compounds are known to show the most potent antioxidant activity, along with other physiological properties such as anti-microbial and anti-inflammatory activity [47,48,49].

To conclude, the results show that both *R. stricta* and *M. crassifolia* exhibited the highest capacity for scavenging DPPH radicals, with IC_50_ values of 335 and 448 µg/mL, respectively. Following the DPPH assay, the H_2_O_2_ scavenging activity of the plant extracts in the cultured HaCaT cells was examined with the ROS-Glo H_2_O_2_ assay, and the findings confirmed that *R. stricta* and *M. crassifolia* showed promising antioxidant activity in both non-cell-based systems and cell culture settings.

## 4. Materials and Methods

### 4.1. Chemicals and Reagents

First, 0.05% Trypsin-EDTA (1X) (Gibco, Sigma-Aldrich Company Ltd., Poole, UK), phosphate-buffered saline (PBS) solution tablets (prepared by dissolving one tablet in 200 mL of deionised water), Dulbecco’s Modified Eagle Medium (Gibco DMEM 1X, Sigma-Aldrich Company Ltd., Poole, UK), containing 4.5 g/L of glucose and 0.11 g/L of sodium pyruvate stored at 4 °C, antibiotics prepared with 100 µg/mL of streptomycin sulphate, 0.25 µg/mL of amphotericin B, and 100 U/mL penicillin G sodium, Gibco foetal bovine serum (FBS), thiazolyl blue tetrazolium bromide (MTT) powder, DMSO, menadione, DPPH (1,1-diphenyl-2-picrylhydrazyl), methanol, fluorescein diacetate (FD), propidium iodide (PI), and ascorbic acid were all purchased from Sigma-Aldrich (Poole, UK). L-glutamine, Gibco trypan blue stain (0.4%), 96-well microplates, 24-well microplates, and fluorescence-activated cell sorting (FACS) tubes were purchased from Sarstedt (Sarstedt Ltd., Nümbrecht, Germany). A ROS-Glo™ H2O2 Assay Kit was purchased from Promega (Promega UK Ltd., Southampton, UK). The antioxidant scavenging activity was measured by the use of a microtiter plate reader (Infinite 200 Pro, Tecan Trading, Männedorf, Switzerland). All other materials were obtained from Thermo Fisher Scientific (Loughborough, UK) and used as received.

### 4.2. Plant Material Collection

Aerial parts of *A. canariense* and *T. macropterus* were collected from the north-western region of Saudi Arabia’s Medina city during March 2017, where a specimen was kept in the herbarium at the Faculty of Pharmacy, King Abdulaziz University under numbers TM 1209 and AC-1131, respectively. Aerial parts of *C. colocynthis* and *M. crassifolia* were collected from the southwestern region of Saudi Arabia’s Al Taif city’s Al Sail Al-Kabeer area during April 2018. Two specimens (CC 1131 and MC 1034) were kept in the herbarium at the Faculty of Pharmacy, King Abdulaziz University. Arial parts of *R. stricta* were collected from the southwestern region of Saudi Arabia’s Al-Shafa mountains of Al Taif city during April 2018. Plant authentication was carried out by the taxonomist Dr. Emad A. Al Sherif, Lecturer in Plant Ecology, Department of Biology, Faculty of Science and Arts, Khulais, King Abdulaziz University, Saudi Arabia. The specimens were deposited in the herbarium of the faculty of pharmacy (RS-1014), King Abdulaziz University.

### 4.3. Plant Extraction

Dried aerial parts of each of *A. canariense*, *C. colocynthis*, *M. crassifolia*, *R. stricta,* and *T. macropterus* were extracted with 8 L of methanol for 72 h by the cold maceration technique (ratio 1 g/10 mL). Each extract was evaporated under reduced pressure to provide total residue yields of 5.59, 8.65, 4.43, 12.18, and 5.26% *w*/*v*.

### 4.4. Cell Culture

Stock solutions of 5 mg/mL were prepared by dissolving 50 mg of plant extracts in 100 μL of 90% methanol; then, 9.9 mL of DMEM was added to make up the final volume of 10 mL, with 1% as a final concentration of methanol. The stock solutions were then filter sterilised and stored at 4 °C.

Culture media were prepared with DMEM, 10% heat-inactivated FBS, 1% L-glutamine, and 1% antibiotics. The seeding density for the HaCaT cells was 1.5 × 10^5^ in a 75 cm^2^ tissue culture flask in an incubator at 37 °C with 5% CO_2_ and 95% humidified air. The medium was replaced with a fresh medium regularly every 24–48 h. To ensure steady cell growth and to reach the required cell density within each 75 cm^2^ tissue culture flask, a sub-culturing procedure was carried out when the cells reached 70–90% confluency.

### 4.5. Antiproliferative Assay: 3-(4,5-Dimethylthiazol-2-yl)-2,5-diphenyl Tetrazolium Bromide Reduction (MTT)

The MTT assay has become the most common cytotoxic and anti-proliferative assay for evaluating test substances [50,51,52]. Following trypsinisation, cells were dispersed with CCM, and the density was set to 5 × 10^4^ cells/mL. Next, 100 µL was added to four 96-well plates and then placed in an incubator. The following day, CCM from each plate was replaced with 100 µL of the serum-free medium, then placed in the incubator at 37 °C, and the sample treatment medium was prepared with DMEM, FBS, L-glutamine, and antibiotics. On the third day, the serum-free medium was discarded from the wells and the test substances were added (*n* = 6). The treatment medium was replaced daily every 24 h. After 24 h (day 1 of the MTT), MTT powder was prepared with PBS, and then 0.2 μm sterile syringe filters were used to sterilise the MTT solution. Next, 25 µL of MTT was added to the day 1 plate, then returned to the incubator with 5% CO_2_ and 95% relative humidity for a duration of 4 h. Following this, the treatment medium, along with MTT, was removed from each well and the formazan crystals were dissolved through the addition of 100 µL of DMSO before being wrapped with clingfilm and placed in the incubator for 30 min. Absorbance at 570 nm was measured using a plate reader (Infinite 200 Pro, Tecan Trading, Männedorf, Switzerland). The MTT assay was carried out in triplicate for each tested plant extract and concentration. The percentage cell growth in the presence of the five plant extracts was determined as follows:

Cell growth % = 100 × (mean absorbance in treatment wells)/(mean absorbance in control wells)(1)

The concentration required to inhibit 50% of HaCaT cell proliferation (IC_50_) was determined using GraphPad Prism 7.0 (GraphPad Software Inc., San Diego, CA, USA).

### 4.6. Fluorescence-Activated Cell Sorting (FACS) Analysis Using Fluorescein Diacetate and Propidium Iodide

To confirm that the HaCaT cell growth inhibition by the plant extracts as shown in the MTT experiment was due to the anti-proliferative action of the extracts rather than cell death, cytotoxic studies of the plant extracts were carried out using fluorescence-activated cell sorting (FACS). This is a specialised type of flow cytometry that is capable of evaluating both viable and non-viable cells through selective staining by PI/FD [53,54]. HaCaT cells were diluted with CCM to achieve a cell density of 5 × 10^4^ cells/mL, and then 1 mL of cell suspension with culture medium was added to a 24-well plate and placed in the incubator at 37 °C with 5% CO_2_ and 95% relative humidity for overnight incubation. The next day, the culture medium was removed from each well and replaced with plant extracts at different concentrations using the same concentrations as those in the MTT assay. Five different controls were prepared in each experiment:

(1) vehicle control: 1% methanol in a cell culture medium.
(2) non-treated, non-stained cell control was included in the experiment to gate the desired population and to calibrate the FACS.
(3) non-treated cells stained with FD only to calibrate living cells.
(4) non-treated cells that were fixed with 200 μL of ethanol for 10 min, and then stained with PI only to calibrate non-viable cells.
(5) non-treated cells stained with both PI/FD to calibrate both stains.

After 24 h, the culture medium from each well was collected in Eppendorf vials to evaluate the dead cells present in the media during the incubation period. Tubes were centrifuged at 500× *g* for 5 min, and then the supernatant was discarded. The attached cells in the 24-well plate were washed twice with 1 mL of PBS, and then 200 μL of trypsin was added and incubated for 10 min; trypsin activity was then neutralised by adding 800 μL of culture medium. Cell suspension from each well was added to the corresponding Eppendorf tube, followed by centrifugation at 500× *g* for 5 min. The supernatant was discarded, and the cells were washed twice with 1 mL of PBS to ensure the removal of the culture medium and the supernatant was discarded before the cell pellet was loosened by gently tapping the tubes. A solution of 300 μL of FD (10 μg/mL in 0.2% DMSO/PBS) and 300 μL of PI (5 μg/mL in PBS) was added to each sample in the Eppendorf tubes. The cells were mixed gently and then placed in the dark for 5 min, before being placed on ice to be analysed within 1 h by flow cytometry (FACSCalibur, Becton Dickinson, Heidelberg, Germany). The system was calibrated with the aforementioned controls, and the fluorescence produced was determined on two separate channels—one for the FD and another for the PI. To set the cells in the most suitable region, the FACS threshold and voltage were adjusted accordingly, and the number of events was set at 20,000. A FACS assay was carried out in triplicate for each tested plant extract and concentration. The percentage of cells stained with FD/PI for the tested plant extracts was determined as follows:

FD/PI stained cells % = 100 × (FD/PI Geo Mean in treatment wells)/ (FD/PI Geo Mean in control wells)(2)

### 4.7. Confocal Laser Scanning Microscopy (CLSM) Analysis Following Live/Dead Staining by Fluorescein Diacetate and Propidium Iodide

Qualitative confirmation of viable and non-viable cells with PI/FD double staining was investigated by confocal microscopy. Following the trypsinisation process, cells were diluted with CCM, and the density was set to 10 × 10^4^ cells/mL. Then, 2 mL of cell suspension was added to a cell culture dish (Mattek 35 mm Dish 1.5 coverslip with a 14 mm glass diameter) and placed in the incubator at 37 °C with 5% CO_2_ and 95% relative humidity for overnight incubation. The following day, the culture medium was removed from each well and replaced with plant extract at the highest concentration tested in the MTT and FACS analyses. On the following day, the culture medium was removed from each dish, a solution of 600 μL of FD (10 μg/mL in 0.2% DMSO/PBS) and 600 μL of PI (5 μg/mL in PBS) was added to each imaging dish. The cells were mixed gently and then placed in the dark for 10 min, following which 1 mL of PBS was used to wash the cells twice, before finally adding 1 mL of imaging medium (DMEM red Phenol free) to each dish before taking the image with CLSM with a Leica SP5 laser scanning microscope equipped with a 65× oil immersion objective. The laser was set at 488 nm for FD and 543 nm for PI. Optimum imaging was gathered by adjusting the gain and offset setting for each experiment. ImageJ software was utilised to process the images gathered from CLSM; the images were split into separate channels and then merged through the software. The image thresholding and conversion to 8-bit allowed optimising the accuracy of each image.

### 4.8. Total Phenolic Content by Folin Ciocalteu (FC) Assay

Folin ciocalteu (FC) assay was used to evaluate the total phenolic content of five plant extracts. Each plant extract was prepared by dissolving 5 mg in 5 mL methanol (1 mg/mL), gallic acid working solutions were prepared in the following concentrations: 0.05, 0.1, 0.15, 0.25, 0.5 mg/mL. Briefly, 0.5 mL of the tested sample was added to a test tube then 2.5 mL of 10% Folin–Ciocalteu reagent was added and the solution was incubated for 8 min, following that 2mL of 20% of a sodium carbonate solution was added to each sample to make up the total volume of 5 mL, the mixtures were mixed then incubated for 2 h in the dark.

Absorbance was measured at 760 nm against a blank solution using a Cary 60 UV/Vis spectrophotometer. Gallic acid calibration curve was generated according to the above-stated concentrations. The results were expressed as mg of gallic acid equivalents GAE/gram of dry plant extract.

### 4.9. Acellular Antioxidant Activity: 1,1-Diphenyl-2-picrylhydrazyl (DPPH)

DPPH at a concentration of 0.2 mM was prepared by dissolving 39.4 mg in 1 mL of methanol, then transferring the solution to a 500 mL volumetric flask and topping it up with the solvent. The solution was covered with aluminium foil and stored at 4 °C. Test solutions were prepared by dissolving it in methanol to a final concentration of 10 mg/mL. The 96-well plate was divided into columns that contain DPPH, columns with the sample, and columns containing the sample and DPPH radical. A serial dilution was carried out for samples in each plate (7.81–1000 µg/mL); then, the plates were wrapped in foil for 30 min, followed by measuring the absorbance by the use of the microplate reader. The percentage of radical scavenging was determined as follows:

% DPPH scavenging = 100 × {(Absorbance Sample+ DPPH) − (Absorbance Sample blank)}/{(Absorbance DPPH) − (Absorbance Solvent)}(3)

The concentration of sample scavenging 50% of the initial DPPH radicals (EC_50_), was determined by interpolating the [(Absorption _sample_) − (Absorption _blank_)] from a DPPH calibration curve [55,56].

### 4.10. Cellular Antioxidant Activity: ROS-Glo H_2_O_2_ Assay with HaCaT Cells

To further evaluate the ability of the plant extracts to scavenge H_2_O_2_, a ROS-Glo™ H_2_O_2_ Kit was utilised, whereby a derivatised luciferin substrate was incubated with the sample and reacted directly with H₂O₂ to generate a luciferin precursor, and the assay was carried out according to manufacturer’s instruction [57].

White 96-well plates with clear bottoms were used to enhance luminescence signals (Greiner Bio-One Ltd., Dursley, UK). HaCaT cells were trypsinised, and then a haemocytometer was used to count the cells; subsequently, the cells were diluted with CCM and the density was set to 10 × 10^4^ cells/mL, before 100 µL of the cell suspension was added to each well. On the next day, the medium was aspirated from each well and 80 µL of the treatment menadione 50 mM, an aromatic ketone compound, acted as a precursor for the synthesis of vitamin K. Menadione, known to generate ROS at high concentrations through a redox cycle [58,59], was added to each well.

Moreover, the following controls were included in each experiment: medium without cells plus vehicle, medium with cells plus vehicle, medium without cells with treatment, positive control menadione (50 µM) with and without cells, and positive control of ascorbic acid (5 µg/mL). Subsequently, 20 µL of the H2O2 substrate dilution buffer (125 µM) was added to make up the final volume of 100 µL in each well, and the plates were then incubated at 37 °C with 5% CO2 for 4 h. Finally, 100 µL of ROS-Glo detection solution was added to the plate and the luminescence signals were recorded using the CLARIOstar plate reader (BMG LABTECH, Offenburg, Germany).

The mean values from the cell-free medium were subtracted from the signals. Data were normalised against the control with cells only and the percentage of H_2_O_2_ reduction was calculated by comparing the treatment to the menadione positive control.

### 4.11. Statistical Analysis

The data obtained were statistically analysed using GraphPad Prism 7.0a (GraphPad Software Inc., San Diego, CA, USA); all data are presented as the mean ± standard deviation (SD) and experiments were performed three times. Excel software (Microsoft) was used for the measurements (means and standard deviations). Statistical analyses were carried out, in which a one-way analysis of variance (ANOVA) with Dunnett’s post-hoc test was utilised to determine statistical significance between the mean of controls and groups, while Tukey’s post-hoc test was used for the statistical significance between groups. A *p*-value < 0.05 was considered significant (* *p* < 0.05, ** *p* < 0.01, and *** *p* < 0.001).

## 5. Conclusions 

In this study, *Citrullus colocynthis* demonstrated cytotoxic effects on HaCaT cells, as shown by the observed significant decrease in cell proliferation from 24h onwards, while *Rhazya stricta* showed significant inhibition of cell growth at 120h and 168h. IC50 values were determined for both plant extracts; for *Citrullus colocynthis*, they were 17.32 μg/mL and 16.91 μg/mL after 5 and 7 days of treatment, respectively; for *Rhazya stricta,* they were 175 μg/mL and 105.3 μg/mL after 5 and 7 days of treatment. Both *Rhazya stricta* and *Maerua crassifolia* showed the highest capacity for scavenging the DPPH radical with an EC50 of 335 and 448 μg/mL respectively, *Rhazya stricta* showed the highest H₂O₂ scavenging activity in the cell-based antioxidant assay. 

## Figures and Tables

**Table 1:** The yield of the different plant extracts following the plant material extraction process with methanol for 72 h.

Extract	Yield
*Aizoon canariense*	5.59%
*Citrullus colocynthis*	8.65%
*Maerua crassifolia*	4.43%
*Rhazya stricta*	12.18%
*Tribulus macropterus*	5.26%

**Table 2:** The IC_50_ of *Citrullus colocynthis*, *Rhazya stricta*, *Aizoon canariense*, *Maerua crassifolia*, and *Tribulus macropterus* were determined from the MTT assay after five and seven days of treatment using the line of best fit. The results were obtained through three independent experiments.

Plant Extract	IC_50_ µg/mL	
	Day 5	Day 7
*Aizoon canariense*	IC_50_ > 200	IC_50_ > 200
*Citrullus colocynthis*	17.32	16.91
*Maerua crassifolia*	IC_50_ > 200	IC_50_ > 200
*Rhazya stricta*	175	105.3
*Tribulus macropterus*	IC_50_ > 200	IC_50_ > 200

**Table 3:** Total phenolic content (TPC) of five plant extracts expressed as gallic acid equivalent per gram of dry powder extract (GAE/g) (mean ± S.D.). Results were obtained through three independent experiments, performed in triplicate.

Extract	TPC (mg GAE/g Dry Extract)
*Aizoon canariense*	7.85 ± 2.48
*Citrullus colocynthis*	37 ± 1.55
*Maerua crassifolia*	24.89 ± 1.06
*Rhazya stricta*	64.04 ± 2.84
*Tribulus macropterus*	21.97 ± 0.74

## Data Availability

The data presented in this study are available on request from the corresponding author.
